# Turning Waste into Greener Cementitious Building Material: Treatment Methods for Biomass Ashes—A Review

**DOI:** 10.3390/ma18040834

**Published:** 2025-02-14

**Authors:** Fatih Bülbül, Luc Courard

**Affiliations:** Urban and Environmental Engineering, University of Liège, Quartier Polytech 1—Allée de la Découverte 9, B-4000 Liège, Belgium; luc.courard@uliege.be

**Keywords:** biomass ash, carbonation, cement, grinding, hydrothermal synthesis, leaching, micro-grinding, substitution

## Abstract

The production of biomass ash (BA) is expected to increase in the future, as biomass is generally considered a carbon-neutral fuel. BA potentially concentrates heavy metals and trace elements at high levels. With the growing production of BA, its disposal in landfills or recycling must be addressed through solid waste policies and within the framework of a circular economy. Utilizing BA as a cement substitute solves disposal issues while offering environmental benefits aligned with the circular economy. However, the varying physical and chemical properties of BA, influenced by factors such as biomass type and combustion technique, necessitate more effective utilization strategies. Consequently, researchers are developing various treatment methods to ensure that BA meet the necessary requirements and do not pose problems such as heavy metal or chlorine leaching. These treatments facilitate the production of concrete with higher compressive strength at greater cement replacement levels, supporting greener construction practices. This review consolidates existing BA data and treatment methods, focusing on their impacts and efficiency. It also explores combined treatments and potential new approaches. By providing a foundation for future research and practical applications, this study aims to improve treatment techniques, helping the industry mitigate environmental risks and advance carbon-neutral construction solutions.

## 1. Introduction

Anthropogenic emissions of CO_2_ come from three main sources: burning of fossil fuels (coal, oil, and gas), large-scale deforestation, and carbonate decomposition [1]. Coal use in energy and power generation is the factor with the largest share in global CO_2_ emissions, with a share of 41% in 2022 [2]. Cement is the largest source of emissions from the decomposition of carbonates [1]. Cement production leads to CO_2_ emissions in two main ways. The first is through chemical reactions during clinker production (5% of total annual anthropogenic emissions), where carbonates (limestone, CaCO_3_) transform into oxides (lime and CaO) and CO_2_ due to heat [1,3,4]. The second source is from fossil fuels or purchased electricity used to heat raw materials in clinker production. Energy-related emissions make up approximately 60% of process emissions [1,5].

**Figure 1:**
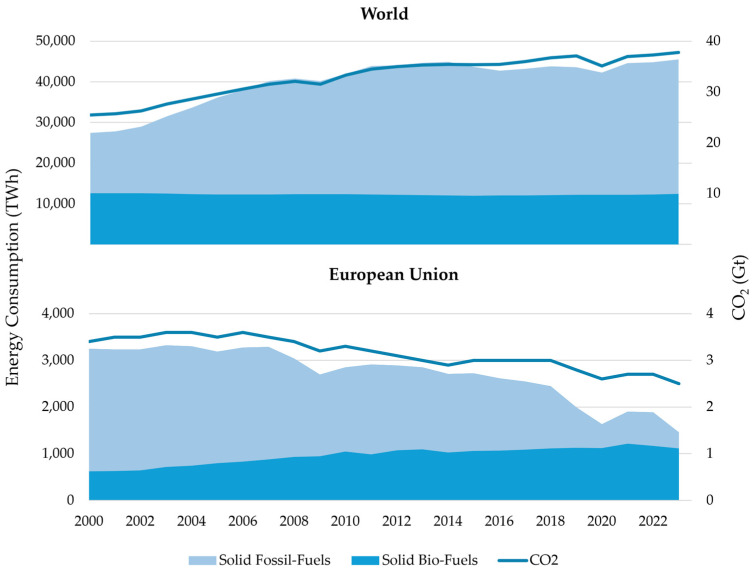
Comparison of solid fossil fuel and solid bio-fuel consumption and CO_2_ emission values for the European Union and the world [2,14,15,16].

Reducing dependence on fossil fuels is crucial for mitigating environmental issues associated with greenhouse gas (GHG) emissions (Figure 1) [6]. It is essential to address the resulting energy deficit by promoting the use of renewable resources in order to secure future energy needs and minimize environmental impacts [7,8,9]. There are various renewable energy sources that can be utilized for energy production [8,10]. Biomass, in particular, garners significant attention due to its widespread availability worldwide and its ease of use in energy production [11]. By 2050, biomass could supply up to 33–50% of the world’s current primary energy consumption [12]. In energy production, biomass is considered carbon neutral because the CO_2_ emitted when burned is equivalent to the CO_2_ absorbed during biomass growth. However, for this to be sustainable, the consumption rate must not exceed the growth rate [13].

Biomass can be defined as a complex heterogeneous mixture of organic and inorganic matter obtained from living or recently deceased organisms and primarily originates from wood sources, as well as from herbaceous agricultural residues like grasses, flowers, straw, husks, and pits [17,18]. Additionally, it encompasses various specific waste streams, including commercial and construction waste like demolition debris and paper pellets, along with household and industrial organic waste, animal and human biomass waste, and aquatic biomass, such as algae [19]. When biomass is mentioned as feedstock for power plants, some biomasses can be seen to be used more widely, such as forest residues, wood chips, agricultural residues, rice husk, wheat straw, municipal solid waste, sewage sludge, and industrial waste [20,21,22]. Additionally, specific to certain geographical locations, biomass types such as sugarcane bagasse, palm oil, olive residue, bamboo leaf, banana leaf, marabou weed, and corn cob can be utilized in energy production [23,24,25,26,27,28,29,30,31,32,33]. By utilizing industrial, municipal, agricultural, and forest waste for energy production, biomass contributes to waste management as well as carbon-neutral energy production.

There are several disadvantages to using biomass in energy production as well as advantages; one of the most important is the generation of solid waste [34]. It is estimated that, all around the world, around 480 million tons of ash are produced from biomass-fired power plants every year [12]. The waste produced by power plants is called biomass ash (BA). There are two types of ash produced in biomass-fueled power plants: fly ash, which consists of lightweight particles carried by exhaust gases, and bottom ash, which consists of heavier unburned or partially burned particles found in the form of slurry or slag [17,34]. BA is a mixture containing complex organic and inorganic materials, including minerals and carbon-based substances in crystalline, semi-crystalline, or non-crystalline phases [8,18].

At present, BA is primarily disposed of in landfills, leading to environmental concerns such as pollution, land degradation, and the aesthetic deterioration of surrounding areas [35,36]. As the amount of ash produced increases, so do the costs of storage [8]. To mitigate these challenges and derive economic benefits, BA has been widely utilized across various industries, including construction, agriculture, environmental remediation, waste management, and the production of ceramics and glass [8,35,37]. Its high alkalinity and surface charge enable the effective adsorption of heavy metals from wastewater, while the presence of unburnt carbon enhances its capacity to capture organic pollutants and flue gas contaminants [38,39,40]. Furthermore, BA serves as a valuable raw material in the production of geopolymers, ceramics, and glass-ceramics, where its mineral composition improves thermal stability and mechanical properties [41,42,43]. Modified BA-based materials have also demonstrated significant potential in phosphate removal from wastewater and mercury capture from flue gases [42,44]. Additionally, the presence of iron oxides and other magnetic minerals expands its applications to metallurgy and ore processing [41,42]. Moreover, BA has been effectively incorporated as a cost-efficient and sustainable binder in backfill materials for mining applications, enhancing their mechanical strength and permeability. By reducing cement consumption in backfill formulations, BA not only improves material performance but also supports resource efficiency and lowers the environmental footprint of mining operations [45,46,47].

In the construction industry, BA is commonly used as a supplementary cementitious material (SCM) in cementitious composites. The high content of minerals such as SiO_2_, Al_2_O_3_, and CaO in BA imparts pozzolanic and hydraulic activity, making it a desirable material. Although numerous studies have demonstrated that BA can positively affect concrete’s strength, durability, thermal conductivity, and acid resistance, it can also contain harmful substances that adversely impact its properties [48,49]. Various treatment methods are employed to reduce the harmful substances in BA and to enhance its reactivity, allowing for higher cement replacement ratios and the production of higher-performance concretes. This study aims to provide a thorough investigation of the treatment methods used for BA, as well as their influence on its characteristics and applications in ash-based concrete. The research presents and compares different treatment techniques, outlining how they modify specific properties and processes. Additionally, it will review the results from prior studies that have employed these methods, offering insights into the effectiveness and outcomes observed. Finally, the study will assess the combined impact of these methods by analyzing findings from earlier research, with a focus on optimizing the performance of BA in concrete applications.

## 2. Properties of Biomass Ash

BA refers to the solid waste produced as a result of the combustion of various types of biomass. Similar to coal ash, BA is composed of fly ash and bottom ash, depending on where the ash accumulates. Biomass fly ash (BFA) is a fine ash fraction that is carried by the flue gases from the biomass combustion chamber and captured by emission control devices such as electrostatic precipitators or bag filters, typically possessing an average particle size ranging from 4 µm to 100 µm, and biomass bottom ash (BBA) is a coarse ash fraction that is accumulated at the bottom of the combustion chamber [50,51].

It is well recognized that even within the same biomass type, factors such as growth conditions, plant maturity, pesticide application, harvest timing, soil characteristics, contamination levels, and the specific plant part utilized can influence ash composition [51,52,53,54,55]. Table 1 presents the minimum, maximum, and average values of major oxides for woody and herbaceous biomasses, highlighting the chemical composition variations between different parts of the same biomass and the effects of filtration and treatment techniques on ash composition. Vassilev et al. [55] demonstrated that BA has the potential to contain elements from nearly the entire periodic table. However, the chemical composition of BA, while showing variability in relative concentrations, primarily consists of SiO_2_, Al_2_O_3_, CaO, MgO, K_2_O, P_2_O_5_, and SO_3_ compounds [12,54,56,57].

The mineralogical composition of BA is characterized by the presence of various secondary mineral phases, including silicates, oxides and hydroxides, sulfates, phosphates, carbonates, chlorides, and nitrates [12,58,59]. The dominant crystalline phases commonly identified in BA include quartz, calcite, sylvite, arcanite, anhydrite, char, glass, lime, periclase, and hematite [12,60]. The mechanisms of silicate formation in BA are quite similar to those in coal ash [61]. Likewise, oxides and hydroxides in BA develop through mechanisms comparable to those in coal fly ash (CFA). These phases primarily emerge from the breakdown and oxidation of organic matter, as well as from the transformation of oxalates, carbonates, phosphates, sulfates, chlorides, and nitrates. Additionally, crystallization of molten material during combustion contributes to the formation of these mineral phases [18]. In contrast, iron compounds such as siderite and hematite have only been observed in forest residue ash [19,60]. Furthermore, some oxy-hydroxides remain unchanged during biomass combustion due to their high melting/decomposition temperatures, persisting as primary minerals in the ash [12].

**Table 1:** Chemical characteristics (main oxides) of BA depending on variables such as biomass type, biomass part, combustion technique, combustion stage, fuel mix, and treatment (forest r.: forest residue).

Ash Type	Main Oxides (%wt)												
	SiO_2_	Al_2_O_3_	Fe_2_O_3_	CaO	P_2_O_5_	K_2_O	MgO	SO_3_	MnO	Na_2_O	Cl	TiO_2_	LoI
Woody biomasses (minimum) [53]	1.86	0.12	0.37	5.79	0.66	2.19	1.10	0.36	-	0.22	-	0.06	-
Woody biomasses (average) [53]	22.22	5.09	3.44	43.03	3.48	10.75	6.07	2.78	-	2.85	-	0.29	-
Woody biomasses (maximum) [53]	68.18	15.12	9.54	83.46	13.01	31.99	14.57	11.66	-	29.82	-	1.20	-
Herbaceous and agricultural biomasses (minimum) [53]	2.01	0.10	0.22	0.97	0.54	2.29	0.19	0.01	-	0.09	-	0.01	-
Herbaceous and agricultural biomasses (average) [53]	33.39	3.66	3.26	14.86	6.48	26.65	4.02	3.61	-	2.29	-	0.18	-
Herbaceous and agricultural biomasses (maximum) [53]	94.48	14.60	36.27	44.32	31.06	63.90	16.21	14.74	-	26.20	-	2.02	-
Wood pellets (spruce) [62]	24.70	5.30	3.20	25.80	4.90	7.90	9.30	-	1.00	2.30	-	0.40	9.70
Pine bark [53]	9.20	7.20	2.79	56.83	5.02	7.78	6.19	2.83	-	1.97	-	0.19	-
Pine chips [53]	68.18	7.04	5.45	7.89	1.56	4.51	2.43	1.19	-	1.20	-	0.55	-
Pine prunings [53]	7.76	2.75	1.25	44.10	5.73	22.32	11.33	4.18	-	0.42	-	0.17	-
Pine sawdust [53]	9.71	2.34	2.10	48.88	6.08	14.38	13.80	2.22	-	0.35	-	0.14	-
Rice straw [53]	77.20	0.55	0.50	2.46	0.98	12.59	2.71	1.18	-	1.79	-	0.04	-
Rice husk [53]	94.48	0.21	0.22	0.97	0.54	2.29	0.19	0.92	-	0.16	-	0.02	-
70% forest r. and 30% peat [63]	52.20	11.00	4.80	16.00	1.70	2.90	3.50	1.70	-	2.10	0.10	-	3.20
60% forest r., 30% recycling waste, and 10% paper sludge [63]	39.40	12.10	4.90	23.00	1.30	2.40	3.10	6.90	-	2.90	0.40	-	0.70
50% forest r. 40% peat, and 10% recycled wood waste [63]	43.80	7.40	2.60	21.10	3.00	6.50	3.40	6.30	-	2.10	0.30	-	5.40
70% forest r. and 30% peat (air-classified) [63]	53.70	11.10	4.70	15.10	1.60	2.90	3.30	1.40	-	2.10	0.10	-	2.90
60% forest r. 30% recycling waste, and 10% paper sludge (air-classified) [63]	49.70	13.60	5.30	14.50	0.90	2.60	2.20	2.40	-	2.90	0.10	-	3.90
50% forest r., 40% peat, and 10% recycled wood waste (air-classified) [63]	56.50	8.80	2.80	13.80	1.80	5.90	2.30	2.20	-	2.20	0.10	-	3.30
Pine sawdust and chips (from wood burning) [64]	9.51	2.67	2.65	5.87	0.89	1.42	1.69	-	0.71	-	-	0.28	74.31
Pine sawdust and chips (from generated ash) [64]	25.06	12.28	8.05	9.90	1.60	3.99	2.60	-	0.69	1.33	-	3.00	31.50
Pine sawdust and chips (from ash disposal) [64]	21.56	10.73	7.82	4.60	0.85	3.03	1.36	-	0.46	0.12	-	1.61	58.59
Wood pellet [65]	3.22	1.07	1.31	42.38	3.23	4.57	4.87	0.59	4.73	0.11	0.07	0.07	-
Wood pellet (calcining and milling) [65]	3.97	1.18	1.44	53.33	5.01	4.19	8.67	0.52	7.20	0.09	0.06	0.09	-
Wood chips (bottom ash) [66]	25.10	4.51	2.28	44.60	4.96	10.20	4.73	-	0.83	0.58	-	0.19	5.40
Wood chips (electrostatic precipitator) [66]	13.50	3.18	1.64	30.90	4.13	18.20	3.70	-	0.41	0.70	-	0.17	21.1
Wood chips (bottom ash) [66]	35.80	0.98	0.79	51.10	3.51	7.31	1.48	-	0.35	0.43	-	0.07	3.51
Wood chips (cyclone filter) [66]	17.90	1.31	8.98	55.00	1.92	1.18	1.27	-	0.31	0.47	-	0.10	30.20
Woody (grate combustion) [67]	11.00	2.40	2.90	53.60	2.90	14.60	4.20	5.40	-	1.00	0.80	-	15.00
Woody (grate combustion—washed) [67]	12.70	3.00	3.20	65.00	3.80	4.40	5.80	1.30	-	1.00	0.00	-	19.60
Woody (circulating fluidized bed) [67]	23.80	5.60	3.10	44.70	3.80	7.60	4.10	6.10	-	0.80	0.40	-	16.20
Woody (circulating fluidized bed—washed) [67]	26.50	6.30	3.30	45.00	4.20	5.70	4.40	3.70	-	0.90	0.00	-	19.70
Wood and peat [68]	41.80	13.10	13.60	16.30	3.50	2.30	2.50	2.10	-	2.10	0.10	0.50	0.30
Wood and peat [68]	30.80	15.10	26.70	12.00	4.90	1.60	2.50	3.50		1.10	0.10	0.40	1.50

Sulfates also play a significant role in the mineralogical composition of BA, formed through interactions between acidic gases and oxides produced during combustion [12]. Additionally, sulfates can form through the interaction of newly generated phosphates with oxides during combustion. Carbonates and amorphous materials (glass) are the final major phases present in BA. Minerals rich in calcium, particularly calcite, anhydrite, and dolomite, are prevalent in BA, which is largely attributed to the naturally high calcium content found in woody biomass [60]. In addition, the formation of an amorphous glassy phase occurs as inorganic components melt during combustion and subsequently solidify upon rapid cooling [12,69,70].

The composition and properties of BA, including its chemical and mineralogical makeup, glass content, unburned carbon levels, elemental distribution, and particle density, are shaped mainly by combustion parameters such as temperature, duration, air-to-fuel ratio, fuel particle size, and combustion rate [12,56,57,59,70]. A study published by Baxter et al. [71] described the properties of BA and its combustion behavior for various biomass fuels. Their study analyzed ash samples from various boiler sections, identifying key inorganic elements like Si, K, Ca, Na, Al, Mg, Fe, S, P, and Cl. Biomass ash formation temperatures vary more widely and are generally lower than coal’s, yet the mechanisms of fundamental phase-mineral transformation show similarities [61]. High Ca, Al, and Ti, along with low contents of K, Si, P, S, Fe, Na, and Mg, contribute to an increased biomass combustion temperature (around 1100 °C). The ash formation process in biomass follows a series of phase-mineral transformations, starting with the generation of intermediate and less-stable compounds such as chlorides, hydroxides, carbonates, sulfates, and phosphates, along with amorphous inorganic materials. This is followed by the development of silicates and glass phases incorporating various salts, eventually leading to the carbonation of newly formed mineral structures. The formation pathways of glass, amorphous phases, char, organic minerals, and other inorganic components are linked to rising combustion temperatures [18]. Specifically, organic material combustion occurs between 200–850 °C, particle fragmentation initiates at 500 °C, particle agglomeration occurs between 700–1300 °C, and mineral fusion progresses in stages: initial melting at 700 °C, extensive fusion between 900 and 1100 °C, complete fusion at 1100–1500 °C, new phase crystallization between 500 and 1500 °C, and molten glass formation between 700 and 1500 °C [18].

During biomass combustion, certain heavy metals volatilize and later condense into the finer fractions of BFA as the material cools [12,72]. This process facilitates the incorporation of elements such as Cd, As, Pb, Ni, Hg, Cr, Cu, Mn, and Zn into the ash matrix, making their presence a significant concern in terms of environmental impact [55]. These potentially hazardous elements typically accumulate in biomass from external sources like soil, pesticides, and fertilizers. Upon combustion, they become mobilized, forming water-soluble compounds in the final BFA. Additionally, combustion conditions influence the unburned carbon content in BFA, the proportion of residual organic material to inorganic matter, and the overall nutrient composition of the ash [52,69].

The physical properties of BA, like its chemical characteristics, vary depending on the type of biomass. The average particle size of BA can range from 0 to 10 mm [49]. Particle size affects the reactivity and workability of the ash. BA also exhibits variations in properties such as density and water absorption. However, it generally tends to have a lower density and higher water absorption compared to inorganic materials like sand and cement. The combustion time and temperature also influence the morphology, texture, and porosity of the ash [73].

## 3. Expectation of Biomass Ash as a Supplementary Cementitious Material

In Europe, the determination of whether BA (non-reactive, non-hazardous, or hazardous) is disposed of in a landfill depends on the leaching concentrations of trace elements [74,75]. BA should be treated as controlled waste in commercial–industrial environments both in the EU and globally [76]. Moreover, contemporary management schemes prefer the recycling of industrial solid waste rather than its disposal [75,76]. Many different articles mention that BA reduces costs for cementitious composites, decrease carbon emissions, provide lower thermal conductivity, and lead to the production of more durable and lightweight elements. This makes BA desirable for SCMs.

BA is utilized as cement substitution, depending on its physical and chemical properties [11,77,78,79,80,81]. The positive effects of using BA as cement substitution on the various properties of cementitious mixtures have been demonstrated in numerous studies [81,82]. However, not all forms of BA exhibit strong pozzolanic reactivity due to their specific chemical and physical characteristics, and some may contain compounds that could be detrimental to cementitious mixtures.

There are currently no established standards for the use of ashes derived from biomass combustion in cementitious mixtures. Due to this lack of specific guidelines, the most practical approach is to apply the standards used for coal ash and natural pozzolans when incorporating biomass ashes into cementitious mixtures. These include standards such as ASTM C618-23 and EN 450-1 [83,84].

**Figure 2:**
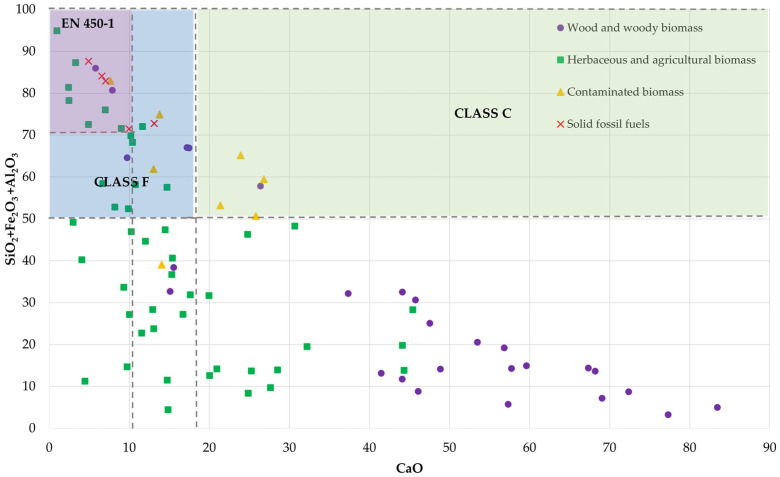
CaO and oxide contents to determine the pozzolanic category of BA [58,85,86,87,88,89,90,91,92,93,94,95,96,97,98,99,100,101,102,103,104,105,106,107,108,109].

Classifying BA according to European and American standards is a complex task, as the two standards have similar yet distinct limitations as shown in Table 2. While some forms of BA, typically classified as Class F, meet the required physical and chemical properties for compliance with these standards, variations in oxide and calcium content often prevent classification in a broad context. These compositional differences pose challenges in aligning different forms of BA with established standards. Based on the 86 types of biomasses identified by Vassilev et al. [53], Figure 2 comparing oxide and calcium contents for woody biomasses, herbaceous and agricultural biomasses, contaminated biomasses, and solid fossil fuels reveals that woody forms of BA generally do not meet the standards. While agricultural forms of BA include more types that align with the standards, a significant portion still fails to comply. For instance, biomass ashes derived from land-clearing wood, pine chips, reed canary grass, sorghastrum grass, rice straw, coconut shells, rice husks, sugarcane bagasse, and mixed wastepaper are considered suitable according to both European and American standards. In contrast, ashes from alder–fir sawdust, Christmas trees, oak wood, miscanthus grass, sweet sorghum grass, switchgrass, wheat straw, and demolition wood meet only the oxide content requirements specified in the American standards.

The use of ashes as SCMs is not only cost-effective and environmentally friendly in terms of the reutilization of ashes and the reduction of clinker production but also contributes to the mechanical and physical properties of products [110]. Research indicates that moderate replacement levels (typically up to 20%) can enhance long-term strength due to pozzolanic reactions, while higher replacement ratios often result in strength reductions due to excessive silica content acting primarily as a filler rather than a reactive component [81]. Studies have shown that compressive strength can be maintained or slightly improved with controlled biomass ash replacement, whereas splitting tensile and flexural strength generally follow similar trends, with moderate reductions at higher replacement levels [111]. However, the contaminants present in the ashes and their varying chemical compositions they possess depend on the type of biomass and combustion process, and their physical properties such as particle size, shape, texture, and surface area must be refined to suit the intended purpose of the cementitious composite. While rearranging these properties through different treatments, limiting factors such as the amount of energy required, the waste produced, time, and cost must be considered to choose the most efficient method.

## 4. Treatments

There are various characteristics of BA that may prevent it from functioning effectively as an SCM. To achieve the desired chemical composition or physical characteristics for their use, certain treatments may be necessary. These treatments can range from low-energy, resource-efficient processes to more complex procedures that require substantial energy and resources. From an environmental perspective, efforts should focus on minimizing additional processing or meeting the desired characteristics through minimal interventions such as screening. High-energy and resource-intensive methods, such as vitrification, chemical washing, or electro-dialytic remediation, which may also lead to extra waste generation, should be carefully evaluated for their performance benefits and economic feasibility. The combination of different improvement techniques has been observed in numerous studies [34,112,113]. In this section, current improvement technologies and cutting-edge processes for utilizing ashes as SCMs will be reviewed.

### 4.1. Grinding

Ensuring that the materials used in concrete have a specific particle size distribution (PSD) is crucial for its overall performance. Therefore, screening aggregates, fillers, or pozzolans to achieve the desired PSD is a common practice. However, the use of materials that do not possess the ideal PSD is also important from the perspective of recycling and the circular economy. Adjusting their particle size for SCMs can significantly enhance pozzolanic reactivity [114]. For ashes that possess suitable chemical properties but contain larger particle fractions, grinding into finer particles can make them more suitable for use [75]. Maschio et al. [115] demonstrated that by grinding and screening BBA, a PSD similar to that of fly ash and cement can be achieved. In a broader context, grinding increases the fineness and surface area of the material, which enhances pozzolanic reactivity and packing density, thereby leading to higher compressive strength [116]. Grinding treatment is particularly effective for agricultural BA, converting highly large porous particles into denser particles [117]. According to the study by Cordeiro et al. [118] on sugarcane bagasse ash, grinding also positively affects concrete resistance to chloride-ion penetration and rheology due to pore refinement resulting from pozzolanic reaction and reduction of the particle interlocking and internal friction, respectively.

According to ASTM C618-23 [83], a good SCM should leave a maximum residue of 34% on a 45 µm (No. 325) sieve. By applying grinding, not only can the actual pozzolanic reactivity of the ashes be enhanced, but compliance with these standards can also be achieved (according to EN 450-1 [84], the maximum ratio is 40%). The duration of the grinding process is also crucial for achieving desired results. Longer grinding times have been observed in various studies to lead to an enhanced surface area and, consequently, higher compressive strength [114,116]. The outcomes of the grinding process are influenced not only by the grinding duration but also by factors such as the type of process, the grinding mechanism, and the speed. These variables can significantly affect the efficiency of treatment. In a study investigating grinding process efficiency, a comparison between vibratory and tumbling mills showed that the vibratory mill produced better results in a shorter time and with less energy consumption [118].

### 4.2. Mechano-Chemical Activation (MCA)

Mechano-chemistry is an extended branch of the grinding process used not only for construction materials but also for processing minerals, food, pharmaceuticals, and chemicals [119]. It is an environmentally friendly process that enables the production of new compounds through solid-state reactions without the use of heat or solvents [120]. Mechano-chemical reactions rely on changes in Gibbs free energy and require mechanical functions such as shearing, compression, and grinding to accumulate the stress energy needed to initiate the reaction [121].

**Figure 3:**
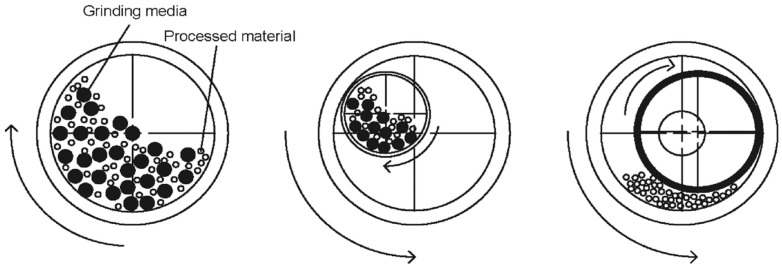
Types of milling equipment processes: **left**—ball mill, **middle**—planetary ball mill, and **right**—ring mill [129].

Mechano-chemical reactions are based on milling, a process that not only reduces particle size but also promotes the formation of reactive surfaces (Figure 3). When precursor materials are crushed between two balls, a significant amount of potential energy is stored, causing bonds to break and leading to the formation of defects and active sites on the surface. The reaction occurs at the interfaces between phases, which makes the creation of fresh and active surfaces essential. Therefore, factors such as the amount of substrate, the size and material of the milling vessel, the number, diameter, and density of the balls, the rotation speed, and the milling time are critical for achieving MCA and ensuring efficiency in ball milling [121]. Depending on these factors, mechano-chemical reactions may not be observed in every grinding process.

MCA, fundamentally a grinding process, results in processed ashes with improved fineness and surface area, leading to similar outcomes in concrete. Grinding reduces particle size and creates new surfaces that serve as precipitation sites for cement hydration products, enhancing the filling effect [122,123,124,125]. The increased pozzolanic reactivity leads to a higher formation of reaction products such as calcium–silicate–hydrate (C-S-H) and calcium–aluminate–hydrate (C-A-H) within the cement matrix. This contributes to a denser microstructure, thereby improving compressive strength [126,127]. MCA can increase the substitution value of non-reactive solid waste, such as BBA without compromising strength by improving their reactivity [112]. This phenomenon was observed in the study of [128]: using untreated fly ash resulted in higher strength compared to the reference mix at a substitution rate of only 10%, whereas milled fly ash allowed for a substitution rate of up to 30%. The factors contributing to this increase include relatively higher contents of SiO_2_ and Al_2_O_3_, lower LOI, and an increase in fineness.

Moreover, MCA can also facilitate the stabilization of heavy metals present in the ash. Chen et al. [130] observed that MCA of municipal solid waste ash (MSWA) resulted in the stabilization of heavy metals such as Cd, Cu, Mo, Pb, Sb, and Zn. When the ash is subjected to prolonged grinding, its microstructure deteriorates, crystallinity decreases, and the structure transforms into amorphous phases resembling a melt-like state, as evidenced by X-ray diffraction (XRD) [128,130]. It is believed that heavy metals are stabilized through absorption by these amorphous phases [124,130,131]. The stabilization of heavy metals suggests that ashes treated with MCA may be suitable for use as SCMs, remaining safe even after weathering and not posing adverse environmental impacts.

The grinding duration and method significantly impact the outcome. As observed in the study by Wu et al. [128], longer grinding increases reactivity and alters the mineralogical structure. MCA reduces the degree of silicate polymerization, and this effect becomes more pronounced as the treatment time extends. The characteristics of BA also vary depending on the grinding method. Due to differences in grinding methods, materials with the same PSD can exhibit different specific surface areas, impacting reactivity and compressive strength. For instance, Wu et al. [128] found that vibration milling resulted in a greater increase in reactivity compared to ball milling.

### 4.3. High-Temperature Treatment (HTT)

HTT of ashes has been observed in various studies, often through processes like re-combustion to eliminate organic matter or vitrification. HTT reduces the LOI and enhances pozzolanic activity [132,133,134]. The feasibility of this treatment depends on the amount of energy consumed and the type of energy source. In a study conducted by [34], HTT was applied by heating BBA to 800 °C and maintaining that temperature for 18 h. Considering the compressive strength improvement achieved, the authors concluded that this treatment was feasible. Other studies have also shown that heating up to 800 °C increases the relative amount of amorphous silica in the ash, enhancing pozzolanic oxides and reactivity [135,136]. This improvement is also due to the removal of volatile compounds and organic matter at around 600 °C [137]. However, beyond 800 °C, pozzolanic reactivity decreases due to the transformation of amorphous silica into crystalline cristobalite [135,136].

In another study, the vitrification process was used to transform ashes into non-crystalline materials with high latent hydraulic reactivity. A mixture of fly and bottom ash obtained from power plants burning both woodchips and straw was used as the ash source. The vitrification process was completed at a temperature of 1500 °C in one hour. The effects of vitrified ash on compressive strength were evaluated against SCMs like limestone, fumed silica, and pozzolan. The results showed that the vitrified ash mortar (at 28 days and 30% replacement) performed better than other SCMs and untreated ash. Performance at 90, 180, and 360 days increased further due to the filler effect, hydration acceleration, and pozzolanic or hydraulic reactions. Additionally, to investigate the effect of vitrification temperature, a second set of samples was heated up to 1300 °C. However, at the lower vitrification temperature, the final product contained crystalline particles, resulting in a glass–ceramic structure and lower performance compared to fully vitrified ash [138].

Vitrification separates harmful organic compounds and stabilizes heavy metals within a glassy matrix [139,140]. It is considered one of the safest methods for processing hazardous waste and transforming it into leach-resistant materials [140]. However, the most prominent disadvantage of vitrification is the high energy consumption required for the melting process [140]. The glassy product obtained through post-vitrification can be used as an SCM, as demonstrated in the study by [138], or as a colored vitreous product, as shown in the works of Ribeiro and Monteiro [139,140].

### 4.4. Hydrothermal Synthesis (HTS)

HTS is a process that uses water under high temperatures and pressure to induce specific chemical and physical changes in various materials. It is commonly employed in waste management, biomass recycling, material synthesis, and mineral processing. HTS is considered as an eco-efficient process due to the fact that it employs low temperatures to activate BA, implying no emission of CO_2_ to the atmosphere, and is increasingly used in the recycling and reuse of industrial waste [141]. Studies on the synthesis of tobermorite from ashes using HTS have a history of about a quarter century, with conducted subtopics including improving process efficiency, temperature and pressure values, starting materials, and the stabilization of harmful elements [142].

Kaminskas et al. [143] investigated the potential use of HTS for synthesizing C-S-H products from BFA and explored the properties of the synthesized material for use as an SCM. In their study, synthesis was carried out at 200 °C for 2 to 24 h. Tobermorite formation was already observed after 2 h of synthesis. The final product contained only quartz, calcite, and tobermorite. Extending the synthesis time to 24 h resulted in an increase in C-S-H products, but this increase was not sufficient relative to the energy expended. According to the results, replacement of up to 10% can be achieved without reducing the compressive strength.

HTS, while utilized for the enhancement of ashes, is also a commonly employed method in the synthesis of zeolites. Although zeolites exhibit a range of mineralogical variations, they can be broadly described as hydrated aluminum-silicate crystals. Depending on their mineralogical variety, zeolites are employed for various applications including water treatment [144,145], the remediation of polluted soils [146], chemical catalysis [147], and as SCMs [148]. Zeolites used as SCMs are typically synthesized from waste products resulting from the production of AlF_3_ [149,150]. When used as SCMs, zeolites generally improve the freeze–thaw resistance and compressive strength of concrete, although they can reduce workability [149,150,151].

Jiménez et al. [141] synthesized tobermorite along with zeolitic phases such as analcime and cancrinite, from a 4 h synthesis process at 200 °C. In their study, NaOH solution was used as the synthesis medium to enable the formation of zeolite and the more efficient dissolution of silica crystals. The reactivity of the treated ash was assessed based on its lime-fixing capacity, showing better performance compared to SCMs like silica fume and metakaolin, as well as untreated ash. Additionally, the absence of chloride ions in the processed ash after HTS is promising for its use as an SCM.

It can be observed from the literature that there is limited research on the use of zeolites obtained from BA through HTS [19]. The study by Pimraksa et al. [148] demonstrated that zeolites produced from CFA can be used as SCMs, showing good performance in both strength and heavy metal stabilization. Fukasawa et al. [152] showed that BA can be employed in the synthesis of zeolites via HTS, suggesting the potential use of these synthesized zeolites as SCMs.

In another study, a BA composite was developed using treated sewage sludge ash (SSA) as a filler, which demonstrated potential for application as a robust material for packaging and insulation purposes. In this composite, straw powder and waste sawdust were utilized as the biomass matrix, with treated SSA serving as the filler. The HTS resulted in the formation of C-S-H products, which enhanced the cohesion performance of the ash as a filler. This improvement enhanced the overall structural integrity and effectiveness of the composite [153].

### 4.5. Washing Treatment

Washing treatment is an easy-to-apply method for improving ashes, involving the washing of ashes with water, solvents, or acids. Washing is an effective method for removing salts such as chlorides, sulfates, and alkaline compounds, as well as heavy metals [154,155]. Since chlorides and sulfates are known to harmfully influence the strength and durability properties of concrete, this treatment could be beneficial for using ash as an SCM.

#### 4.5.1. Water Washing (WW)

WW can be carried out in a single step or in several sequential steps. Several studies have demonstrated that chlorides can be effectively removed in the initial step of washing [156]. According to these studies, WW is a viable method for chloride removal. However, the authors of [156] noted that the efficiency of removal decreases in subsequent steps. Phua et al. [157] reported that WW can achieve up to 90–95% removal of chlorides from the ash, improving the quality of the final product for further utilization. Nevertheless, Yang et al. [158] have raised concerns about the potential risk of heavy metal leaching in discharge waters. According to Zhu et al. [159], the use of chemical additives and pH adjustment could address this issue.

#### 4.5.2. Chemical Washing (CW)

There is limited research on washing ashes with solvents or acids. CW is generally applied for the removal of heavy metals [160]. Washings with sulfuric acid and phosphoric acid have shown good results in the removal of heavy metals such as Pb, Zn, and Cu [159]. In the study by Ma and Zhang [161], phosphoric acid was found to be more effective than sulfuric acid. Washing treatment with HCl is also quite effective in removing Pb, Cd, Cu, and Zn [160]. Pöykiö et al. [162] investigated the leaching behavior of residues in ashes using a three-step washing process with water, ammonium acetate (CH_3_COONH_4_), and HCl solutions, respectively. They found that water extraction was effective for S, ammonium acetate extraction was effective for Cu and S, and HCl extraction was effective for the removal of Ti and Al concentrations.

In washing processes aimed at reducing the chlorine content of ashes, factors such as the liquid-to-solid ratio, stirring speed, temperature, and time play a significant role [161,163]. For instance, a higher liquid-to-solid ratio yields better results. However, there is currently no standard or general consensus on washing parameters [164]. The washing process also has some drawbacks, including the large volumes of water required and the necessity to treat process waters for contamination before discharge [154,155,159,161,163].

### 4.6. Electro-Dialytic Remediation (EDR)

**Figure 4:**
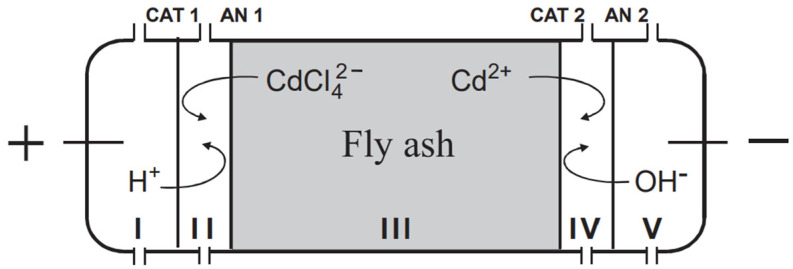
The principle of EDR (CAT: cation exchange membrane, AN: anion exchange membrane) [169].

EDR is a method that combines electro-dialysis with the electro-migration of ions to remove the mobile fraction of heavy metals from ash, thereby reducing heavy metal and salt leaching. The fundamental principle of the EDR involves placing a solution containing an ash suspension into electro-dialytic cells. An electric current is applied across the cells, facilitating the transport of metal ions to the electrodes according to their charges. Ion-exchange membranes placed between the cells enable the collection of metal ions before they reach the electrodes and subsequently their removal from the solution as shown in Figure 4 [164]. In EDR, the ion transfer medium can be deionized water, NH_3_ solution, citric acid solution, or ammonium citrate solution [165,166,167,168].

According to the study by [165], the EDR technique is not very efficient in the removal of heavy metals. Ebbers et al. [166] suggest that efficiency can be improved by using certain ancillary agents to optimize heavy metal removal. However, this can result in the production of a more challenging liquid waste that requires further treatment before reuse or disposal [170]. Additionally, EDR is a method that requires continuous energy consumption and is quite time-consuming [164].

Although EDR has been used in several studies for the removal of Cd from BA, these studies did not aim to use the treated ash as an SCM [169,171]. According to the study conducted by [169], EDR was able to remove 70% of the Cd present in the ash. In another study conducted by Chen et al. [171], EDR reduced the leachability of heavy metals such as Se, As, Ba, Pb, Cu, Zn, Cd, Cr, and Mn (except Ni). Researchers noted that the reduction in K content during the process also decreased the fertilizer value of the ash. However, the ash can still be used as an SCM or as a clay substitute in bricks [172,173]

In the study conducted by Kirkelund et al. [174], which is one of the few studies investigating the use of EDR-treated ash as an SCM, it was found that the heavy metal leaching of MSWA mortars was similar to that of the reference mortars without any additives. The compressive strengths were also comparable to those of CFA mortars (at 28 days with 15% replacement, achieving 40 MPa). However, it was observed that the initial setting time of treated ash mortars increased up to 16 h and workability decreased. According to Kirkelund et al. [174], due to its high salt content, treated ash should only be considered for unreinforced concrete.

### 4.7. Carbonation—Accelerated Carbonation (AC)

Under specific conditions, CO_2_ can be absorbed and stabilized in alkaline solid waste, such as BA, in the form of carbonate minerals or dissolved carbonate ions, depending on the alkalinity and reactivity of the waste [112,175,176]. AC is used to reduce the environmental impact of waste materials and improve their structural properties [177]. The primary goal of this treatment is to facilitate the binding of CO_2_ by reactive components within the ash, thereby reducing the amount of carbon in the atmosphere. AC of ashes represents a sustainable and environmentally friendly approach to CO_2_ sequestration. During AC, CO_2_ reacts with compounds like calcium silicate and aluminate to form calcium carbonate (CaCO_3_). The presence of phases such as lime, free CaO, and portlandite in the material facilitates CO_2_ binding, while carbonation of silicate phases like gehlenite and akermanite is more challenging [178]. Additionally, a finer particle size enhances carbonation [179]. Carbonation also stabilizes the chemical structure of the ash and reduces leaching risks.

The use of carbonated ashes in mortars has been investigated by various researchers. While carbonated ashes may lead to reduced compressive strength due to their larger particle size, they can produce more durable products resistant to dissolution and chemical degradation, thanks to their more stable chemical structure [112]. Previous studies have shown that carbonated waste, when used as SCMs, provides higher strength compared to untreated waste and reduces Ca leaching [180,181,182].

### 4.8. Air-Classification Treatment (ACT)

**Figure 5:**
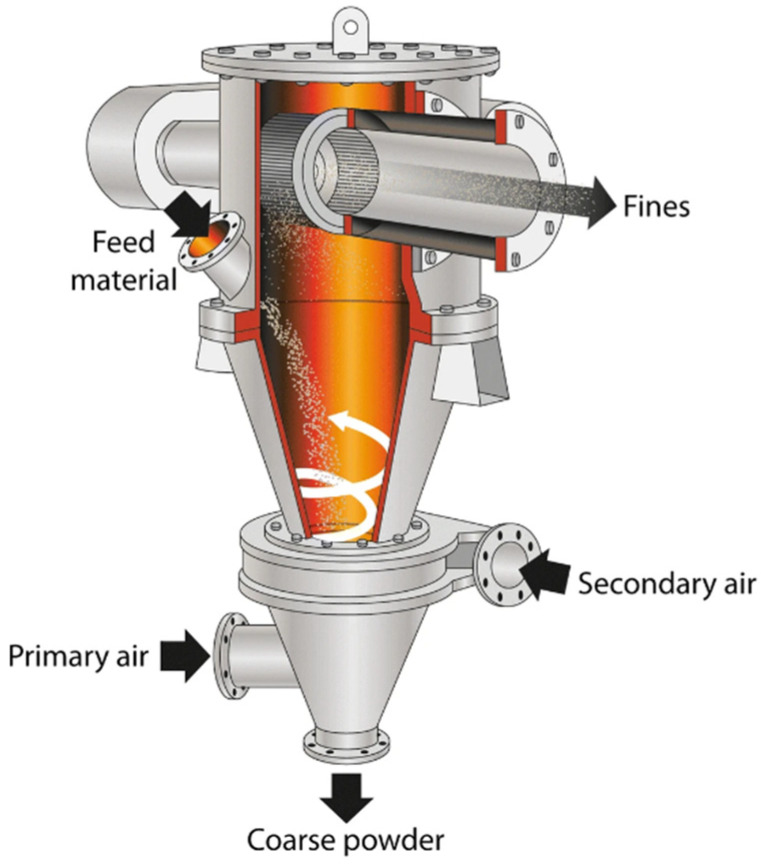
Working principle and classification procedure of the air-jet classifier [183].

ACT is a method used to analyze the ash in a dry state based on particle size and to remove detrimental materials such as sulfates, chlorides, heavy metals, or unburnt carbon found in fine fractions [72,183]. ACT essentially involves exposing the ash to airflow at a specific speed, where the finer ash fractions are captured and separated by the airflow while the coarser ash fractions pass through the airstream and are deposited separately (Figure 5). The process is highly dependent on airflow and requires different speeds for different materials. In the study conducted by Ohenoja et al. [183], after ACT, it was found that the SiO_2_ and Al_2_O_3_ fractions remained in the coarser ash fraction, while harmful substances such as sulfates, chlorides, and heavy metals (Cd, Cu, Zn, and Pb), along with the CaO fraction, were retained in the finer ash fraction. By separating the finer fraction from the ash, a material that met standards for sulfate and chloride content was obtained. Similarly, De La Grée et al. [113] found that ACT was highly effective in removing fine carbon particles and the chlorides and sulfates attached to these particles.

Zhang et al. [184] observed in their study that the fine fraction obtained after ACT was rich in amorphous phases, as well as chlorides, sulfates, and unburnt carbon, while the coarse fraction was abundant in crystalline phases. Through pozzolanic activity and compressive strength tests, they found that fractions with particle size below 8 μm exhibited higher reactivity and were deemed more suitable for use as SCMs. Another study by Ohenoja et al. [63] provided data consistent with these findings. The ash subjected to ACT showed lower reactivity compared to their previous studies. The researchers attributed this to the removal of Ca and S content along with the fine fractions during the ACT process.

### 4.9. Combined Treatments

There are various treatment processes applied to improve the properties of BA for its use as an SCM. In the literature, it is often observed that these treatment methods are combined. By using different thermal, chemical, and mechanical treatments together, the adverse effects of one treatment can be mitigated by another, ash quality can be improved, and more eco-efficient and cost-effective processes can be developed.

Rosales et al. [34] aimed to determine the optimal treatment process for BA by applying three different treatment methods and their combinations to mitigate the adverse effects of organic matter on the durability and mechanical properties of cementitious mixtures. The methods used in the study include combustion (800 °C for 18 h), flotation, and grinding (to a particle size finer than 125 µm), as well as their combinations. All treatments successfully reduced organic matter content; however, combustion demonstrated the greatest efficiency, while grinding showed the lowest efficiency. The lowest organic matter content was achieved with the combination of all treatments, while the contribution of flotation and grinding treatments to combustion was only 3%. The lowest porosity and water absorption values were also obtained with the triple-combined treatment. For both porosity and water absorption, grinding proved to be the most effective treatment. Similarly, for compressive and flexural strength results, the triple-combined treatment yielded values closest to those of mortar without ash, with grinding being the most effective treatment. Table 3 presents the compressive strength values from studies investigating different treatment methods for biomass ashes from various sources. The grinding-combustion combination achieved 75% and 100% increases in strength at 20% and 38.5% replacement ratios, respectively. However, the final strength at the 38.5% replacement remained lower compared to the 20% replacement. According to the researchers, grinding and combustion processes are considered feasible for using BA as an SCM. However, flotation is theoretically applicable but considered a challenging and expensive process.

De La Grée et al. [113] conducted a study aimed at reducing the leaching of contaminants from BA. To achieve this, the researchers examined the effectiveness of various treatment techniques, both individually and in combination, for ashes with different physical and chemical properties, and designed a combined treatment approach. The methodology involved initially removing carbon particles from the ashes. Subsequently, the ashes were washed to reduce the content of soluble salts (such as chloride and sulfate), heavy metals, and aluminum and finally ground to achieve a suitable PSD to enhance reactivity. The study authors found that washing alone was insufficient for chloride removal in some ashes. The researchers hypothesized that soluble chlorides were bound to the surface of carbon particles and thus did not dissolve in water. To address this issue, they proposed two pre-treatments to remove carbon particles before washing: First, re-heating the ashes to 750 °C to burn off the carbon particles, and second, applying air-filtration to physically separate fine carbon particles from the ash mixture. Following these pre-treatments, the chlorine content in the ashes decreased by 70% and 75%, respectively, supporting the researchers’ hypothesis. With the removal of carbon content, the total reduction in chlorine content for ashes subjected to re-washing was 93% and 82%, respectively. Sieving the ashes also improved chlorine reduction in some cases. In experiments with ashes containing large carbon particles, washing without sieving achieved a chlorine removal rate of 71%, while sieving through a 500 µm sieve before washing increased this rate to 91%. Air-filtering and re-heating processes, which removed fine carbon particles, led to changes in the PSD of the ashes. Ashes with increased average particle size were ground to meet the standard PSD. Based on experimental data, the researchers developed a pilot process for scaling up the washing procedure to an industrial scale, incorporating cycloning, flotation, and rinsing stages. According to the results of the study, an industrial-scale treatment method was developed that is both cost-effective and eco-efficient compared to ash storage.

In a study aimed at developing waste-derived SCMs, Skevi et al. [112] applied a combination of MCA and carbonation treatment to BA. The study authors found that with a 40% replacement ratio, the compressive strength of mortar with BA was only 17% of that achieved without any ash addition, whereas mortar with mechano-chemically activated biomass ash (MBA) reached 63% of the reference strength. The 28-day compressive strength of mortar with 40% MBA (23.17 MPa) was nearly identical to that of mortar with 20% untreated BA (25.01 MPa), which is promising for more economical and eco-efficient concrete. MCA led to a decrease in the intensity of akermanite and the formation of the carbonate phase baylissite (K_2_Mg(CO_3_)_2_·4H_2_O), with new carbonates forming due to surface defects and atmospheric CO_2_. Other observed changes included the amorphization of calcites and an increase in amorphous silica content in the ash. Replacing 40% of cement with carbonated MBA resulted in a 28.5% increase in 28-day compressive strength compared to the reference, which was significantly lower than the values for MBA mortars but nearly twice that of mortars containing untreated ash. The carbonation process led to the formation of stable carbonate phases in the MBA. Consequently, the active Si-O bonds on the surface of the carbonated MBA likely bonded with CO_2_ during the carbonation process, leaving fewer active sites compared to MBA. The formation of carbonates may have also contributed to the larger particle sizes of carbonated MBA, which could reduce the filling effect compared to MBA. Although the carbonation of BA before incorporation into cementitious composites adversely affected compressive strength, the potential to reduce the carbon footprint to near zero is a significant outcome. Therefore, it is recommended to maintain relatively low replacement levels in applications involving carbonation to avoid substantial reductions in strength.

ACT is an effective method for removing harmful substances from the fine fraction of ash. However, as demonstrated in several studies, the method yields better results when combined with grinding [183,184]. In the study of Ohenoja et al. [183], prior to ACT, ash was subjected to grinding solely to eliminate agglomerations formed during storage and transportation, which facilitated the separation of the fine fraction and its associated contaminants. A drawback of the ACT is that it can adversely affect the PSD of the ash. In their study, De La Grée et al. [113] used combined treatments, whereby they re-ground the ash after ACT, achieving the desired PSD in ash containing fewer detrimental materials. A similar result was obtained in the research by Ohenoja et al. [63], where ACT significantly reduced the levels of SO_3_ and Cl in the ash, while the content of “SiO_2_ + Al_2_O_3_ + Fe_2_O_3_” relatively increased. Additionally, the median particle size increased from approximately 45 µm to 200 µm, and to rectify this, grinding was employed, reducing the median particle size to 30 µm. This resulted in an improvement in both flexural and compressive strength.

## 5. Conclusions

The production of biomass ash as a residue of biomass combustion is increasing worldwide. The chemical and mineral composition of biomass ash is extremely varied, and a general evaluation of its utilization cannot truly be made. The integration of biomass ash into concrete has demonstrated significant potential in contributing to both improved material performance and more sustainable construction practices. Biomass ash contains beneficial components such as silica, alumina, and iron oxides, which contribute to its reactivity. Depending on its chemical composition and fineness, untreated biomass ash can sometimes be directly used as a supplementary cementitious material, though its performance may vary considerably. Nevertheless, the effectiveness of biomass ash as a supplementary cementitious material is highly dependent on the treatments applied to enhance its properties, such as particle size distribution, reactivity, and contaminant content.

It is important to note that certain properties can be improved depending on the treatment methods used, while others may be adversely affected. Heavy metal and chlorine content may decrease, but particle size distribution may deteriorate, reducing the filler effect. Alternatively, while reactivity and fineness may increase, some contaminants might remain in the system, reducing the durability of the mixture. Therefore, careful optimization of these treatments is necessary to balance the beneficial and adverse impacts specific to the chemical and physical properties of the ashes, ensuring that the overall performance of the concrete is enhanced. Moreover, the environmental advantages of using biomass ash are considerable, as its inclusion in concrete production can reduce the reliance on traditional cement, thereby lowering greenhouse gas emissions and conserving natural resources.

Considering these findings, the effective treatment and use of biomass ash should be further explored to maximize its potential as a key component in sustainable construction. In line with this goal, future research conducted by our team will focus on examining the effects of the mechano-chemical activation method on biomass ashes obtained from different sources, as this review identifies mechano-chemical activation as one of the most effective treatment methods. This approach could provide valuable insights into enhancing the sustainability and durability of concrete structures.

## Figures and Tables

**Table 2:** Characteristics of ashes to be used in concrete applications according to EN 450-1 and ASTM C 618-23 [83,84].

Characteristics	EN 450-1 (%wt)	ASTM C618-23 (%wt)
LOI	A ≤ 5–B ≤ 7–C ≤ 9	≤6
SiO_2_ + Al_2_O_3_ + Fe_2_O_3_	≥70	≥50
Chloride	≤0.1	-
Sulfate (SO_3_)	≤3	≤5
Free CaO	≤1.5	-
Reactive CaO	≤10	F≤18–C>18
Reactive SiO_2_	≥25	-
Moisture Content	-	≤3
Total Alkalis (Na + K)	≤5	-
MgO	≤4	-
P_2_O_5_	≤5	-
Fineness (45 μm)	S ≤ 12–N ≤ 40	≤34
Activity Index (7 day)	-	≥75
Activity Index (28 day)	≥75	≥75
Activity Index (90 day)	≥85	-

**Table 3:** Effects of treatments methods on the strength properties of different ashes [34,63,67,112,185].

Ash Type	Source	Treatment	Substitution Ratio (%)	Reference Strength (MPa)	Untreated Strength (MPa)	Treated Strength (MPa)	Effect of Treatment
BBA	Wood and timber combustion	Mechano-Chemical Activation	40%	36.80	6.24	23.17	+271.31%
		Mineral Carbonation and MCA	40%	36.80	6.24	10.48	+67.95%
BBA	Wood (whole tress and primarily pine)	Sieving–Washing	20%	69.90	51.10	59.30	+16.05%
BFA	70% forest residues and 30% peat	Classification	20%	49.40	25.80	29.00	+12.40%
		Classification + Grinding	20%	49.40	25.80	36.60	+41.86%
BFA	60% forest residues, 30% recycling waste, and 10% paper sludge	Classification	20%	49.40	27.80	29.20	+5.04%
		Classification + Grinding	20%	49.40	27.80	34.20	+23.02%
BFA	40% peat, 50% forest residues, and 10% recycled wood waste	Classification	20%	49.40	31.40	36.60	+16.56%
		Classification + Grinding	20%	49.40	31.40	38.40	+22.29%
BBA	Olive tree prunings	Washing	20%	58.03	25.12	27.61	+9.91%
		Heat Treatment	20%	58.03	25.12	29.12	+15.92%
		Grinding	20%	58.03	25.12	36.18	+44.03%
		Washing + Heat Treatment	20%	58.03	25.12	33.21	+32.21%
		Washing + Grinding	20%	58.03	25.12	38.92	+54.94%
		Heat Treatment + Grinding	20%	58.03	25.12	44.17	+75.84%
		Washing + Heat Treatment + Grinding	20%	58.03	25.12	51.96	+106.85%
BFA	Wood chips	Washing	15%	46.50	40.00	38.00	−5.00%
		Washing	30%	46.50	38.20	39.00	+2.00%

## Data Availability

No new data were created in this study.
